# Metabolic Reversal of Functional Hypogonadism? GLP-1 Receptor Agonists and Male Reproductive Endocrinology—A Systematic Review

**DOI:** 10.3390/cells15151319

**Published:** 2026-07-23

**Authors:** Zakhi Zafrani, Sarah Suleman Khan, Michael Zitzmann

**Affiliations:** 1 Independent Researcher, Manchester SK8 6HD, UK; 2 Department of Clinical and Surgical Andrology, Centre of Reproductive Medicine and Andrology, Münster University Hospital, 48149 Munster, Germany; profzitzmann@gmail.com

**Keywords:** testosterone deficiency, GLP-1 receptor agonists, functional hypogonadism, obesity, metabolic health, male reproductive endocrinology

## Abstract

**Highlights:**

**What are the main findings?**

- GLP-1 receptor agonists are associated with modest increases in testosterone in men with obesity- or diabetes-associated functional hypogonadism.
- In healthy eugonadal men, these agents do not significantly alter reproductive hormones or semen parameters.

**What are the implications?**

- Endocrine responses to GLP-1 therapies appear phenotype-dependent and largely mediated by improvements in metabolic health.
- GLP-1 receptor agonists may represent a fertility-preserving metabolic strategy in selected men with functional hypogonadism.

**Abstract:**

Background: Testosterone deficiency is highly prevalent in men with obesity and type 2 diabetes mellitus and often reflects functional suppression of the hypothalamic–pituitary–gonadal (HPG) axis. Glucagon-like peptide-1 receptor agonists (GLP-1 RAs) are widely used in these conditions, but their effects on male reproductive endocrinology remain incompletely defined. Objective: To systematically review the effects of GLP-1 receptor agonists on testosterone, HPG axis hormones, and male reproductive outcomes. Methods: A systematic review was conducted in accordance with PRISMA 2020 guidelines. PubMed/MEDLINE, Cochrane, and Google Scholar were searched (January 2021–January 2026) for studies evaluating GLP-1 RA therapy in adult men with reported endocrine or reproductive outcomes. Results: Eight studies met inclusion criteria, including one randomised placebo-controlled crossover trial and six observational or interventional studies. In men with obesity, type 2 diabetes, or functional hypogonadism, GLP-1 RAs were associated with modest increases in testosterone and improvements in erectile function or selected semen parameters. In contrast, a placebo-controlled trial in healthy eugonadal men showed no significant endocrine or reproductive effects. Responses appeared dependent on baseline metabolic status. Conclusions: GLP-1 RAs are associated with improvements in male reproductive endocrinology in metabolically compromised populations with metabolic hypogonadism while remaining neutral in healthy individuals. Larger prospective studies are needed.

## 1. Introduction

Testosterone plays a critical role in numerous physiological processes, spanning endocrine, metabolic, psychological, and general aspects of male health. Glucagon-like peptide-1 receptor agonists (GLP-1 RAs) are incretin-based agents commonly prescribed for obesity and type 2 diabetes (T2DM), with emerging evidence and interest regarding their potential direct or indirect influence on circulating testosterone levels.

Testosterone deficiency (TD) is highly prevalent amongst men with obesity and T2DM, with epidemiological studies indicating that up to 40–50% of men within these populations meet biochemical criteria for hypogonadism [1,2]. Recent endocrine consensus statements increasingly conceptualise obesity-associated testosterone deficiency as a functional and potentially reversible suppression of the hypothalamic–pituitary–gonadal (HPG) axis rather than a primary testicular disorder [3,4]. In this model, metabolic disturbances, including insulin resistance, visceral adiposity, chronic inflammation and altered adipokine signalling, impair hypothalamic GnRH pulsatility and Leydig cell steroidogenesis, resulting in secondary hypogonadism despite structurally intact testes [5,6,7,8,9].

Clinically, TD in men with obesity and T2DM is associated with adverse cardiometabolic profiles, reduced lean mass, increased visceral adiposity, impaired insulin sensitivity, sexual dysfunction, fatigue, and diminished quality of life [1,10,11]. While testosterone replacement therapy (TRT) remains effective for symptomatic men with confirmed biochemical deficiency, its role in functional hypogonadism remains debated due to concerns regarding long-term safety, fertility suppression, and the potential to overlook reversible metabolic drivers [12].

Lifestyle modification, bariatric surgery and improvements in insulin sensitivity have consistently been associated with partial restoration of endogenous testosterone production [13,14,15]. GLP-1 RAs, including liraglutide and semaglutide, are now cornerstone therapies for obesity and T2DM due to their potent effects on weight reduction, glycaemic control, and cardiovascular risk reduction [16,17]. Emerging evidence suggests that GLP-1 RAs may also exert favourable effects on male endocrine function, positioning them at the intersection of metabolic and androgen health [18,19].

Obesity-associated functional hypogonadism is best understood as a reversible suppression of the hypothalamic–pituitary–gonadal (HPG) axis driven by a constellation of metabolic disturbances [3,4]. Visceral adiposity promotes aromatisation of androgens to oestrogens, increasing negative feedback on hypothalamic GnRH secretion [8]. Insulin resistance impairs Leydig cell steroidogenesis and reduces hypothalamic insulin signalling [7]. Chronic low-grade inflammation—characterised by elevated interleukin-6, tumour necrosis factor-alpha, and C-reactive protein—further suppresses GnRH pulsatility and gonadotropin release [5,6]. Altered leptin and adipokine signalling disrupt the metabolic cues that sustain normal HPG axis function [5,9]. Concurrently, reduced hepatic production of sex hormone-binding globulin (SHBG) in the context of insulin resistance results in low total testosterone even when free testosterone may be comparatively less affected [3]. These mechanisms collectively create a phenotype of functional, potentially reversible hypogonadism that is mechanistically distinct from classical primary or secondary hypogonadal disorders [3,4].

Within this context, GLP-1 receptor agonists represent a particularly interesting therapeutic class because they simultaneously influence weight, insulin sensitivity, inflammation and cardiovascular risk factors. Consequently, their potential endocrine effects may reflect systemic metabolic restoration rather than direct stimulation of androgen production. This paradigm has important therapeutic implications. Whereas classical hypogonadism requires hormone replacement, metabolic hypogonadism may improve through interventions targeting the underlying metabolic drivers [13,14]. The relationship between T2DM and functional hypogonadism is bidirectional: testosterone deficiency worsens insulin resistance and promotes visceral adiposity, while metabolic dysfunction suppresses HPG axis activity—creating a self-reinforcing cycle that may be interrupted by effective metabolic therapy such as GLP-1 RA treatment.

## 2. Methods

This systematic review was conducted and reported in accordance with the PRISMA 2020 guidelines [20].

### 2.1. Information Sources and Search Strategy

The search strategy was developed iteratively and piloted to ensure sensitivity and reproducibility. A comprehensive literature search was conducted to identify studies evaluating the effects of glucagon-like peptide-1 receptor agonists (GLP-1 RAs) on male reproductive endocrinology. Electronic databases searched included PubMed/MEDLINE, the Cochrane Central Register of Controlled Trials (CENTRAL), and Google Scholar. The search covered the period from 1 January 2021 to 31 January 2026. The search window was restricted to January 2021 onwards to capture the modern GLP-1 RA and dual-incretin era, during which reproductive and endocrine endpoints in men began to be systematically reported alongside metabolic outcomes in clinical studies. Earlier literature was considered in the background section but was not included in the systematic search, as GLP-1 RA use for obesity with male reproductive endpoints as pre-specified outcomes is a post-2020 phenomenon.

The search strategy combined Medical Subject Headings (MeSH) and free-text terms related to incretin-based therapies and male reproductive outcomes. Key search terms included “GLP-1 receptor agonist”, “semaglutide”, “liraglutide”, “dulaglutide”, “exenatide”, “tirzepatide”, “dual incretin”, “testosterone”, “hypogonadism”, “testosterone deficiency”, “male reproductive hormones”, “spermatogenesis”, “semen parameters”, and “fertility”. Boolean operators (AND, OR) were used to combine search terms appropriately. Searches were restricted to studies involving human participants, published in English, and involving adult male populations.

The full electronic search strategy for PubMed/MEDLINE is provided to ensure reproducibility in accordance with PRISMA 2020 guidelines. The PubMed search strategy was adapted for use across the other databases (see Appendix A).

To maximise completeness, additional search methods were employed. Reference lists of included studies and relevant review articles were manually screened to identify further eligible studies. Abstracts from major scientific meetings, including those of the Endocrine Society (ENDO), the European Association of Urology (EAU), and the International Continence Society (ICS), were also reviewed to capture relevant unpublished or emerging data.

All records identified through database and manual searches were imported into reference management software, and duplicate entries were removed prior to screening.

This review was registered with the International Prospective Register of Systematic Reviews (PROSPERO) (Registration number: CRD420261370402). It was conducted according to a pre-specified registered protocol, and no major deviations occurred.

### 2.2. Eligibility Criteria

We included human studies published in English between January 2021 and January 2026 that evaluated adult men (aged ≥ 18 years) treated with GLP-1 RAs. Eligible studies included men with functional hypogonadism, particularly in the context of obesity and/or type 2 diabetes. Studies were required to include a GLP-1 RA intervention (e.g., liraglutide, exenatide, semaglutide, or other agents within the class) and to report at least one endocrine or reproductive outcome of interest, including serum testosterone, luteinising hormone (LH), follicle-stimulating hormone (FSH), or semen parameters. Both randomised controlled trials (RCTs) and non-randomised studies, including observational and cohort studies, were eligible. Comparators included placebo, no treatment, or alternative interventions, including TRT.

Controlled or prospective pilot studies evaluating combined GLP-1 RA or dual incretin plus testosterone replacement strategies were also considered eligible, provided they reported the endocrine and reproductive outcomes specified above.

Studies were excluded if they were animal or in vitro studies, case reports, editorials, expert opinions, systematic reviews, or bibliographic reviews. Studies without a GLP-1 RA treatment arm or an appropriate comparator were also excluded. Additional exclusion criteria included studies involving men with primary testicular disorders (e.g., Klinefelter syndrome, testicular tumours, or severe varicocele), organic hypothalamic or pituitary disease (e.g., prolactinomas or hypopituitarism), or the use of medications known to significantly affect the HPG axis, such as opioids or other hormonal therapies. Non-English-language publications were excluded.

### 2.3. Outcomes

The primary outcome of this systematic review was the change in serum total testosterone levels before and after treatment with a GLP-1 RA. Where available, both baseline and post-intervention testosterone concentrations were extracted and analysed.

Secondary outcomes included additional endocrine and reproductive parameters. Endocrine outcomes comprised changes in free testosterone, sex hormone-binding globulin (SHBG), LH, and FSH. Reproductive outcomes included changes in semen parameters, such as sperm concentration, total sperm count, motility, morphology, and semen volume, when reported.

### 2.4. Study Selection and Data Extraction

**Figure 1:**
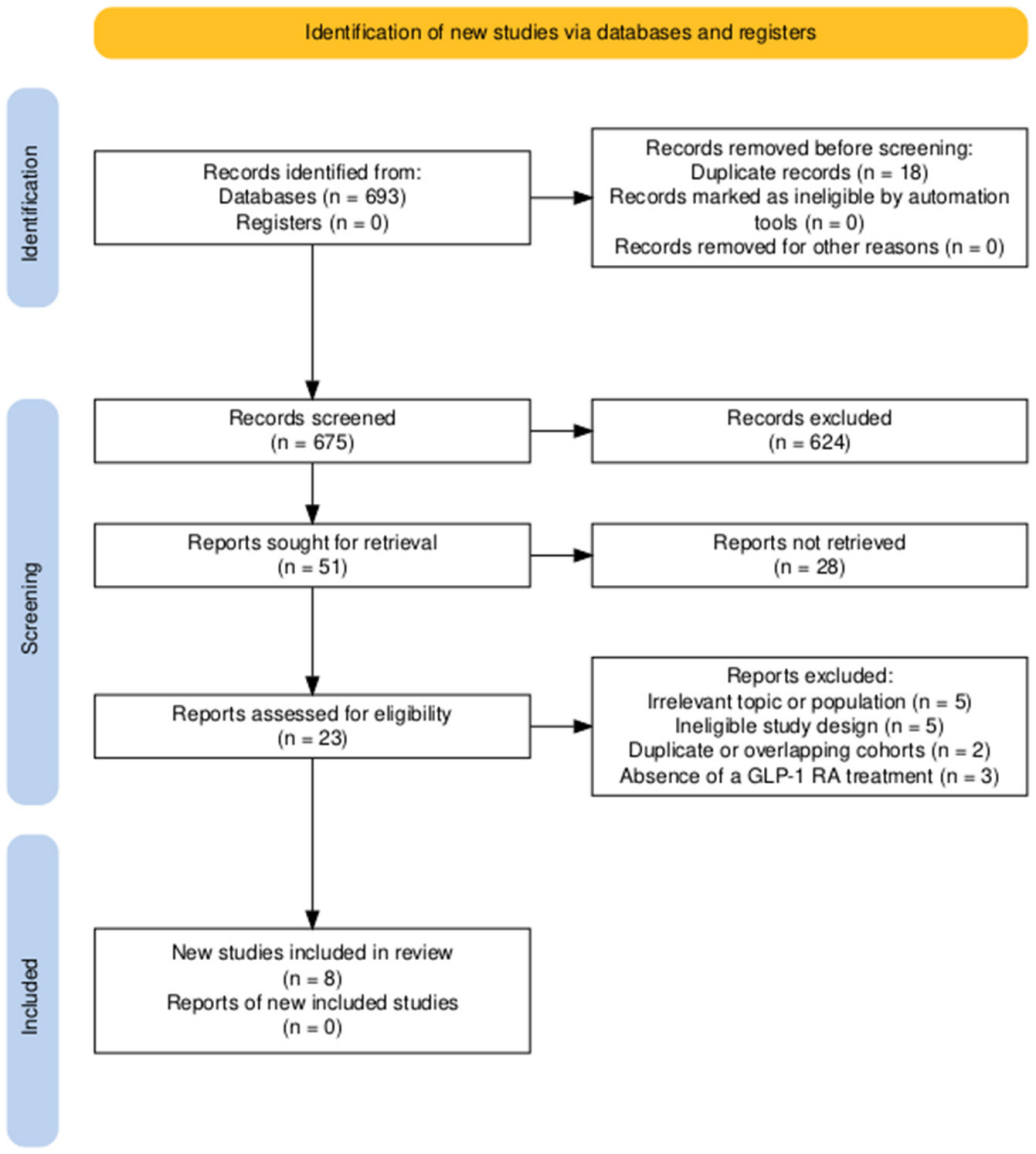
PRISMA flow diagram [21].

After initial deduplication, all records identified through database searching and manual conference screening were imported into a reference manager. Titles and abstracts were screened for relevance, followed by full-text review of potentially eligible studies. Reasons for exclusion at the full-text stage were documented. The study selection process is summarised in a PRISMA 2020 flow diagram (Figure 1).

Data extraction was performed independently by two reviewers using predefined protocols. Extracted variables included study design, sample size, population characteristics, GLP-1 RA used, duration of therapy, baseline metabolic status, endocrine outcomes (total testosterone, free testosterone, SHBG, LH, FSH), reproductive outcomes (semen parameters where available), and key findings.

Discrepancies in data extraction were resolved through discussion and consensus. Where necessary, study authors were consulted or Supplementary Materials reviewed to clarify reported outcomes.

Agreement between the two reviewers during title/abstract screening and full-text selection was assessed through independent parallel screening followed by discussion. Disagreements were resolved by consensus between the reviewers. No formal Cohen’s kappa statistic was calculated for this review; this is acknowledged as a methodological limitation, and future systematic reviews in this area should prospectively report inter-rater reliability using validated measures.

Upon full-text assessment, 15 studies were further excluded as they did not meet the predefined inclusion criteria, since several studies that appeared relevant based on title and abstract did not ultimately report male endocrine or reproductive outcomes, lacked a GLP-1 receptor agonist intervention arm, included mixed-sex populations without sex-specific analyses, represented duplicate cohorts, or employed ineligible study designs.

### 2.5. Risk of Bias Assessment

The risk of bias of included studies was assessed independently by two reviewers using validated tools appropriate to the study design. Randomised controlled trials were evaluated using the Cochrane Risk of Bias tool (RoB 2), and non-randomised studies, including cohort and observational studies, were assessed using the Risk Of Bias In Non-randomised Studies of Interventions (ROBINS-I V2) tool. Discrepancies between reviewers were resolved through discussion and, when necessary, consultation with a third reviewer.

## 3. Results

### 3.1. Study Selection

The study selection process is summarised in the PRISMA 2020 flow diagram (Figure 1). A total of 693 records were identified through database searching and manual screening of conference abstracts. After removal of 18 duplicate records, 675 records were screened based on title and abstract. Of these, 624 records were excluded as they did not meet the predefined eligibility criteria. The full texts of 51 articles were sought for retrieval; however, 28 records could not be retrieved in full text despite multiple access attempts and were excluded from the final synthesis. The remaining 23 full-text articles were assessed for eligibility, of which 16 were excluded for not meeting the inclusion criteria. Exclusions were categorised by reason as follows: irrelevant topic or population (*n* = 5; including female-only cohorts, paediatric populations, or studies reporting only non-endocrine outcomes); ineligible study design (*n* = 5; including case reports, editorials, and narrative reviews without primary data); absence of a GLP-1 RA treatment arm or appropriate comparator (*n* = 3); and duplicate or overlapping cohorts already represented by an included study (*n* = 2). Ultimately, eight studies were included in the qualitative synthesis (Table 1).

### 3.2. Study Characteristics

The main characteristics of the eight studies included in this systematic review are summarised in Table 1. The studies were published between 2021 and 2026 and included adult male participants aged ≥18 years. Study designs comprised one randomised, double-blind, placebo-controlled crossover trial, one prospective cohort study, two retrospective cohort studies, and three controlled or pilot interventional studies, including a prospective pilot study evaluating combined dual incretin and testosterone therapy in late tirzepatide responders. Across all seven included studies, a total of approximately 1200 adult male participants were enrolled, though the exact aggregate number varies depending on how overlapping cohorts are counted (individual study sample sizes are reported in Table 1). Studies spanned multiple countries and healthcare settings, reflecting diverse clinical populations with obesity, T2DM, or functional hypogonadism.

The study populations varied across investigations. Four studies enrolled men with obesity and/or T2DM and functional hypogonadism, one study focused on obese fertile men with functional hypogonadism, and one study included healthy men without known endocrine disease. Sample sizes ranged from small pilot cohorts to larger observational populations.

A range of GLP-1 receptor agonists were evaluated, including exenatide, liraglutide, dulaglutide, semaglutide, and tirzepatide. Treatment duration varied between studies, ranging from short-term interventions to longer observational follow-up periods. Comparators included placebo, no treatment, or baseline pre-treatment assessments, depending on study design.

Endocrine outcomes were reported in all included studies, with serum total testosterone assessed universally. Several studies also reported additional hormonal parameters, including free testosterone, SHBG, LH, and FSH. Reproductive outcomes were evaluated in a subset of studies and included erectile function measures and semen parameters, such as sperm morphology, concentration, motility, and semen volume. Serum total testosterone was measured by immunoassay or liquid chromatography–tandem mass spectrometry (LC-MS/MS), depending on study centre; testosterone deficiency was variously defined across studies as total testosterone below 10.4, 12.0, or 14.0 nmol/L, reflecting heterogeneity in the reference thresholds applied. Sexual function was assessed using the International Index of Erectile Function (IIEF or IIEF-5), a validated psychometric questionnaire, in the studies that reported sexual outcomes.

Overall, there was substantial heterogeneity in study design, patient populations, GLP-1 RA agents, outcome measures, and follow-up duration, which precluded quantitative meta-analysis and formal calculation of a pooled heterogeneity statistic (I^2^) across studies. A narrative synthesis was therefore conducted. Qualitatively, the direction of effect was consistent across studies enrolling men with metabolic disease (testosterone increased or was unchanged with a trend toward increase), while the placebo-controlled trial in healthy eugonadal men demonstrated no effect, indicating a phenotype-dependent pattern of response. The magnitude of testosterone change varied substantially across studies, reflecting high clinical and methodological heterogeneity.

### 3.3. Risk of Bias

Across the seven included studies, one randomised controlled trial was assessed as having low risk of bias using the Cochrane RoB 2 tool. The remaining six non-randomised studies were assessed using the ROBINS-I tool and demonstrated moderate to serious risk of bias overall, primarily due to confounding, small sample sizes, and non-randomised allocation of interventions (Table 2 and Table 3).

Outcome measurement bias was generally low, as testosterone and reproductive outcomes were objectively assessed in most studies. However, heterogeneity in study design and incomplete adjustment for metabolic confounders limited the overall certainty of evidence.

### 3.4. GRADE Summary

The overall certainty of evidence was assessed using the GRADE approach (Table 4). Across outcomes, the certainty of evidence ranged from very low to moderate. Evidence for changes in total testosterone was rated as low certainty, primarily due to serious risk of bias in non-randomised studies, small sample sizes, and imprecision. Similarly, evidence for free testosterone, SHBG, and erectile function outcomes was rated as low to very low certainty, reflecting heterogeneity in study design, variability in outcome reporting, and limited controlled data.

Semen parameters demonstrated very low certainty of evidence, largely due to small sample sizes, inconsistent measurement methodologies, and high between-study heterogeneity. In contrast, metabolic outcomes such as weight reduction and glycaemic control demonstrated moderate certainty, supported by more consistent findings across multiple study designs and larger sample populations.

Overall, the certainty of evidence was limited by reliance on observational data, short follow-up durations, and the lack of testosterone and reproductive outcomes as primary endpoints in most studies. Further high-quality randomised controlled trials are required to strengthen the evidence base and clarify causal relationships.

### 3.5. GLP-1 Receptor Agonist Effects

Across the seven included studies, several consistent patterns emerged despite methodological heterogeneity. First, GLP-1 receptor agonists were generally associated with modest increases in circulating testosterone in men with metabolic disease and functional hypogonadism. Second, these endocrine changes were frequently accompanied by improvements in metabolic parameters, including glycaemic control and body composition. Third, reproductive outcomes, particularly erectile function and selected semen parameters, tended to improve or remain stable during GLP-1 RA therapy. In men with obesity, insulin resistance, T2DM, or functional hypogonadism, GLP-1 RAs were associated with increases in total testosterone and, in several cases, free testosterone concentrations. These associations were not observed in healthy eugonadal men, supporting a phenotype-dependent pattern of endocrine response that appears to reflect metabolic restoration rather than direct androgen stimulation.

Importantly, the only placebo-controlled crossover trial conducted in healthy eugonadal men demonstrated no significant changes in reproductive hormones or semen parameters during dulaglutide exposure [22]. Taken together, these observations suggest that the endocrine effects of GLP-1 receptor agonists are context-dependent and primarily manifest in metabolically compromised populations, consistent with the concept of reversible metabolic hypogonadism.

An important mechanistic question concerns whether the observed testosterone increases reflect a direct effect of GLP-1 receptor activation on gonadal function or are secondary to improvements in insulin sensitivity, body weight, and systemic inflammation. Preclinical studies have identified GLP-1 receptor expression in Leydig cells, Sertoli cells, and spermatozoa, raising the possibility of direct gonadal modulation [6,7]. However, no clinical study has yet demonstrated a direct GLP-1 receptor-mediated effect on testosterone secretion independent of concurrent metabolic improvement. In the studies included in this review, testosterone increases were consistently accompanied by improvements in glycaemic control, weight, and insulin resistance, and were entirely absent in the placebo-controlled trial in metabolically healthy eugonadal men. This pattern strongly implicates indirect metabolic mechanisms—particularly improvements in insulin sensitivity and reductions in visceral adiposity—as the primary drivers of HPG axis reactivation, consistent with the established relationship between insulin resistance and Leydig cell steroidogenesis [7]. Future studies using GLP-1 RA administration with rigorous control for weight loss are required to disentangle direct from indirect gonadal effects.

### 3.6. Adjunct Testosterone Therapy in Persistent Hypogonadism After Incretin Treatment: Preliminary Evidence Only

One prospective pilot study extended these observations to a distinct and clinically relevant subgroup. Ten obese men with functional hypogonadism who failed to achieve ≥5% total body weight loss after ≥3 months of tirzepatide (classified as late responders) were allocated to tirzepatide monotherapy or tirzepatide plus intramuscular testosterone undecanoate 1000 mg for 6 months. Tirzepatide alone resulted in progressive lean body mass (LBM) loss and did not restore testosterone or sexual function. By contrast, the addition of testosterone markedly altered the clinical trajectory: LBM increased to 66.1 ± 3.1 kg versus 63.4 ± 3.0 kg (*p* < 0.01), IIEF-5 scores improved to 23.2 ± 2.1 versus 18.0 ± 1.5, HOMA-IR decreased to 2.9 ± 0.6 versus 3.8 ± 0.7 (*p* < 0.01), and physical activity nearly doubled.

These preliminary findings suggest that, in a carefully selected subgroup of late tirzepatide responders with persistent functional hypogonadism, adjunct testosterone therapy may offer additional clinical benefit. However, the very small sample size (*n* = 10) and non-randomised allocation preclude definitive conclusions; these results should be considered hypothesis-generating pending confirmation in larger controlled trials.

## 4. Discussion

### 4.1. Functional Hypogonadism as a Metabolically Driven Endocrine Disorder

This systematic review examined the effects of GLP-1 receptor agonists on male reproductive endocrinology across seven clinical studies published between 2021 and 2026. The principal finding is that GLP-1 RAs are associated with modest but consistent increases in circulating testosterone in men with obesity, insulin resistance, or T2DM-associated functional hypogonadism, while exerting no significant endocrine effect in healthy eugonadal men. Secondary findings include improvements in erectile function, preservation of spermatogenesis, and—in one small pilot study—additional benefit from adjunct testosterone therapy in men with persistent hypogonadism despite incretin-based treatment. The following discussion contextualises these findings within existing mechanistic and clinical evidence.

The concept of functional hypogonadism has gained increasing recognition in recent years. Unlike classical forms of hypogonadism caused by primary testicular failure or structural hypothalamic–pituitary disease, functional hypogonadism reflects reversible suppression of the HPG axis induced by systemic metabolic disturbances. Obesity, insulin resistance, chronic inflammation and altered adipokine signalling collectively impair hypothalamic GnRH secretion and Leydig cell steroidogenesis [3,9]. In this context, circulating testosterone concentrations often correlate with indices of metabolic health rather than with intrinsic testicular pathology.

This distinction is clinically relevant because therapeutic strategies targeting metabolic dysfunction may restore endogenous androgen production without the need for lifelong testosterone replacement therapy. Improvements in weight, insulin sensitivity and inflammatory signalling have repeatedly been associated with increases in testosterone levels in obese men [13,14]. The studies analysed in the present review support this paradigm by demonstrating that GLP-1 receptor agonists were associated with increases in total testosterone and partial improvement of the testosterone profile primarily in men whose HPG axis is suppressed by metabolic disease, while exerting little or no endocrine effect in healthy eugonadal individuals.

These findings support the concept that, in obesity-associated functional hypogonadism, improvement of metabolic health may partially restore endogenous HPG-axis activity. Across retrospective cohorts, prospective interventional studies, randomised controlled trials, and controlled crossover designs, GLP-1 RAs—and more recently, dual incretin agonists—demonstrate the potential to improve testosterone levels and reproductive function in metabolically compromised men, while remaining hormonally and reproductively neutral in healthy eugonadal individuals. This is consistent with the evolving concept of functional hypogonadism as a potentially reversible condition driven by metabolic dysfunction rather than irreversible gonadal disease [3,4]. However, the current evidence remains heterogeneous and largely non-randomised; firm conclusions about clinical practice should await larger prospective controlled trials.

### 4.2. Phenotype-Dependent Testosterone Responses

A consistent finding across the included studies is that the testosterone-modulating effects of GLP-1-based therapies are phenotype-dependent. In men with obesity, insulin resistance, T2DM, or functional hypogonadism, GLP-1 RAs are associated with increases in total and, in some cases, free testosterone. Conversely, in lean, metabolically healthy men, these agents do not significantly alter HPG axis hormones. The randomised, double-blind, placebo-controlled crossover trial by Lengsfeld et al. demonstrated that dulaglutide did not affect testosterone, gonadotropins, sexual desire, or sperm parameters in healthy men, supporting the notion that GLP-1 RAs do not act as supraphysiological stimulants of the HPG axis [22].

In metabolically compromised populations, observational and interventional studies demonstrated more consistent endocrine benefits. Lisco et al. reported significant improvements in total and free testosterone over 12 months in men with T2DM and erectile dysfunction treated with long-acting GLP-1 RAs, whereas testosterone levels declined in men treated with metformin alone, indicating that improved glycaemic control alone is insufficient to explain endocrine recovery [23]. Similarly, Eid et al. reported a mean increase of approximately 111 ng/dL in total testosterone in men with obesity and/or T2DM treated with GLP-1 RAs, alongside improvements in metabolic parameters [24]. These findings align with earlier epidemiological and mechanistic studies linking metabolic dysfunction to suppression of testosterone production [1,2,5].

The prospective observational study by Graybill et al. adds nuance to this phenotype-dependent effect. In 51 men with T2DM treated with exenatide extended-release for six months, overall changes in total and free testosterone were not statistically significant. However, men with baseline total testosterone concentrations below 320 ng/dL or those achieving meaningful reductions in HbA1c experienced significant increases in testosterone [25]. Importantly, these changes were not associated with modest weight loss, suggesting that baseline hormonal status and metabolic improvement—rather than absolute weight reduction—are key determinants of endocrine response.

Taken together, the available evidence is consistent with the hypothesis that GLP-1-based therapies improve testosterone predominantly through reversal of metabolic suppression of the HPG axis. Whether direct GLP-1 receptor activation at the level of the gonad contributes independently to these effects remains uncertain; no clinical study has yet demonstrated a direct GLP-1 receptor-mediated effect on testosterone secretion in the absence of concurrent metabolic improvement. These findings should therefore be interpreted as evidence of metabolic restoration rather than direct androgenic stimulation.

An apparent paradox exists in that GLP-1 RAs may increase testosterone in obese men with functional hypogonadism while reducing androgen excess in obese women with polycystic ovary syndrome (PCOS). This is best understood not as a sex-independent androgenic or anti-androgenic drug effect but as metabolic normalisation operating within different endocrine contexts. In obese men, visceral adiposity, insulin resistance, inflammatory signalling, altered leptin/adipokine dynamics, and low SHBG contribute to functional suppression of GnRH/LH signalling and Leydig cell steroidogenesis. By improving weight, insulin sensitivity, hepatic SHBG production, and systemic inflammation, GLP-1 RAs may relieve this suppression and permit partial recovery of endogenous testosterone production. In women with PCOS, by contrast, insulin resistance amplifies ovarian theca-cell androgen production and lowers SHBG, thereby increasing free androgen exposure. GLP-1 RA-induced improvements in insulin resistance and body weight reduce insulin-driven ovarian androgen excess and increase SHBG, leading to reductions in free testosterone or the free androgen index. Thus, GLP-1 RAs do not exert a uniform pro- or anti-androgenic action; rather, they tend to move reproductive endocrine function toward the sex- and phenotype-specific physiological set point. This mechanistic framing resolves the apparent paradox and reinforces that the endocrine effects of GLP-1 RAs are best interpreted in terms of metabolic context rather than a direct hormonal action.

### 4.3. Mechanisms: Metabolic Restoration Rather than Direct Endocrine Stimulation

The available evidence indicates that testosterone improvements are mediated predominantly through reversal of metabolic suppression of the HPG axis. Weight loss, reductions in visceral adiposity, improved insulin sensitivity, and attenuation of chronic low-grade inflammation are established drivers of testosterone recovery in obesity-associated hypogonadism [5,7,9]. Increases in SHBG, reported in studies of liraglutide and tirzepatide as well as by Graybill et al., further suggest hepatic and metabolic normalisation. However, the dissociation between total and free testosterone in some cohorts highlights the importance of distinguishing between biochemical and bioavailable androgen recovery.

Several findings challenge a purely weight-dependent mechanism. Eid et al. and Gregorič et al. observed no significant correlation between weight loss and testosterone improvement [24,26], and Lisco et al. reported that testosterone changes did not independently predict improvements in erectile function [23]. These dissociations suggest additional contributory mechanisms, including improved hypothalamic insulin signalling, altered leptin and adipokine dynamics, and potential direct testicular effects. Preclinical studies have identified GLP-1 receptor expression in Leydig cells, Sertoli cells, and spermatozoa, supporting a possible role for local gonadal modulation, although the relative contribution of direct versus indirect effects remains unclear [6,7].

Dual incretin agonism may amplify these mechanisms. The controlled pilot study by La Vignera et al. demonstrated that tirzepatide induced rapid increases in total, free, and bioavailable testosterone, alongside rises in LH and FSH, suggesting robust reactivation of endogenous gonadal function that may exceed the effects of GLP-1-only therapies [27].

### 4.4. Sexual Function: Permissive Rather than Primary Testosterone Effects

GLP-1 RA-based therapies consistently improved erectile function and sexual desire in men with metabolic disease, although testosterone recovery appears to be a permissive rather than primary driver. In the study by Lisco et al., GLP-1 RA exposure itself was the strongest predictor of erectile function improvement, followed by weight loss and glycaemic control, whereas testosterone change was not independently predictive [23]. These findings suggest pleiotropic benefits mediated through improved endothelial function, nitric oxide bioavailability, and reduced inflammation—mechanisms known to contribute to sexual dysfunction in obesity and T2DM [10,11].

Nevertheless, the sexual benefits observed with GLP-1-based therapies, often comparable or superior to those reported with TRT, underscore the clinical value of restoring endogenous androgen production within a metabolically optimised environment rather than relying solely on exogenous testosterone [12].

The study by Seminara et al. adds further nuance to this relationship. In men with persistent hypogonadism despite treatment with tirzepatide, monotherapy did not significantly improve sexual function, as reflected by stable IIEF-5 scores. However, the addition of testosterone replacement therapy resulted in a clinically significant improvement, restoring scores to the normal range [28]. These findings indicate that in individuals who fail to achieve endogenous hormonal recovery, direct androgen supplementation may be necessary to realise functional benefits.

This suggests that GLP-1-based therapies improve sexual function primarily through metabolic and vascular mechanisms, with testosterone acting as a permissive factor that enhances, but does not independently drive, clinical outcomes.

### 4.5. Fertility and Spermatogenesis

A key distinction between GLP-1 RAs and exogenous testosterone lies in their effects on fertility. Across available studies, GLP-1 RAs preserved or improved spermatogenesis while maintaining gonadotropin secretion. Gregorič et al. demonstrated that semaglutide modestly increased testosterone while preserving LH and FSH and improving sperm morphology, in contrast to TRT, which suppressed gonadotropins and reduced sperm counts [26]. Similarly, La Vignera et al. reported that liraglutide improved semen parameters and outperformed both gonadotropin therapy and TRT in obese fertile men with functional hypogonadism [15]. These findings are consistent with the well-established fertility-suppressive effects of exogenous testosterone [12].

### 4.6. Body Composition and Sarcopenia

A consistent concern across the literature is the potential for lean body mass (LBM) loss during GLP-1 RA-based therapy, particularly in hypogonadal men who are already at increased risk of sarcopenia. While some authors have proposed combining GLP-1 RAs with testosterone replacement therapy (TRT) in selected patients, emerging evidence suggests that the impact of incretin-based therapies on body composition is phenotype-dependent rather than uniformly deleterious [24].

In metabolically responsive populations, studies of tirzepatide demonstrate concurrent reductions in fat mass alongside preservation or even gain of lean mass, likely mediated by improvements in insulin sensitivity and restoration of endogenous testosterone production [27]. These findings challenge the assumption that GLP-1-based therapies inevitably exacerbate sarcopenia and instead suggest that favourable body composition changes can occur in individuals who achieve sufficient metabolic and hormonal recovery.

In men with persistent hypogonadism, Seminara et al. reported that tirzepatide monotherapy led to progressive lean body mass loss over six months, indicating a potential risk of sarcopenia in this subgroup. However, the addition of TRT not only prevented further LBM loss but resulted in a clinically significant recovery of lean mass, accompanied by a marked increase in physical activity levels [28]. This dual effect likely reflects both the direct anabolic actions of testosterone on skeletal muscle and indirect behavioural influences, including enhanced energy, motivation, and exercise capacity.

These findings reinforce that the effects of GLP-1-based therapies on lean mass are contingent on baseline hormonal and metabolic status. In men who fail to achieve endogenous testosterone recovery, combined therapy with TRT may be required to mitigate sarcopenic risk and optimise functional outcomes, highlighting the importance of incorporating LBM and physical activity as key endpoints in future studies.

### 4.7. Relationship to Testosterone Replacement Therapy

An important clinical question is how GLP-1 receptor agonist therapy should be positioned relative to conventional testosterone replacement therapy (TRT). TRT remains the standard treatment for men with classical hypogonadism and persistent symptomatic testosterone deficiency. However, in obesity-related functional hypogonadism, endocrine guidelines increasingly emphasise addressing underlying metabolic drivers before initiating lifelong androgen therapy [12].

The findings summarised in this review suggest that GLP-1 receptor agonists may represent a complementary and, in some cases, upstream therapeutic strategy in selected patients. By improving metabolic health and facilitating partial restoration of endogenous hypothalamic–pituitary–gonadal (HPG) axis activity, these agents may help reverse endocrine dysfunction while avoiding the suppression of gonadotropin secretion and spermatogenesis associated with exogenous testosterone administration. This distinction is particularly relevant in younger men wishing to preserve fertility.

Nevertheless, current evidence remains limited, and GLP-1 receptor agonists should not be considered direct substitutes for TRT. Rather, they are best viewed as components of an integrated metabolic–endocrine treatment strategy. Preliminary evidence from Seminara et al. suggests that in a subset of men—those identified as late incretin responders with persistent hypogonadism—the addition of TRT to dual incretin therapy may represent a rational, targeted approach [28]. However, because TRT suppresses endogenous LH and FSH secretion as well as spermatogenesis, it is not appropriate for men seeking to preserve fertility, and careful patient selection with thorough counselling is essential.

### 4.8. Limitations

Several limitations of this systematic review should be noted. The number of included studies is small (*n* = 7), reflecting the nascent state of the literature. Study designs are heterogeneous, ranging from a randomised placebo-controlled crossover trial to retrospective cohort analyses, limiting direct comparison. Different GLP-1 RA agents, doses, and treatment durations were used across studies, and it remains unclear whether endocrine effects are class-wide or agent-specific. Follow-up durations were generally short, and the durability of testosterone recovery is unknown. Semen parameter data were reported inconsistently and in very small samples, precluding robust conclusions about fertility outcomes. Most studies did not report free testosterone or SHBG consistently, making it difficult to determine whether bioavailable androgen concentrations improved to the same degree as total testosterone, an important distinction given that SHBG rises with weight loss and may elevate total testosterone independently of free androgen status. The relative contributions of direct GLP-1 RA effects on the gonad versus indirect effects mediated by weight loss and metabolic improvement cannot be separated in most included studies. No formal meta-analysis was possible due to outcome heterogeneity. Finally, the absence of a formally calculated Cohen’s kappa for inter-rater reliability is acknowledged as a methodological limitation of this review.

### 4.9. Clinical Implications and Future Directions

Collectively, these studies support reframing functional hypogonadism as a metabolically driven, potentially reversible condition. GLP-1 RAs and dual incretin agonists target upstream pathophysiology rather than simply replacing testosterone. In men with obesity or diabetes and associated hypogonadism, particularly those wishing to preserve fertility, GLP-1-based therapies may represent a first-line or adjunctive strategy, with TRT reserved for persistent symptoms or refractory biochemical deficiency [3,12].

Important evidence gaps remain. Most studies are short-term, sample sizes are modest, testosterone is rarely a pre-specified primary endpoint, and reproductive outcomes are inconsistently reported. The study by Seminara et al. further highlights the importance of precision phenotyping: identifying men who are late GLP-1 or dual incretin responders with persistent hypogonadism may guide the rational use of combined pharmacological strategies [28].

Future trials should include LBM, physical activity, and sexual function as co-primary endpoints alongside testosterone and should prospectively evaluate the durability of combined incretin and TRT approaches in this distinct metabolic phenotype. Furthermore, they should also clarify the long-term durability of hormonal and reproductive improvements, optimal patient selection and combination strategies, mechanistic contributions of direct versus indirect gonadal effects, and comparative efficacy across GLP-1-only and dual incretin agonists.

## 5. Conclusions

In summary, emerging evidence indicates that GLP-1 receptor agonists are associated with increases in total testosterone and partial improvements in reproductive endocrine parameters in men with obesity- or T2DM-associated functional hypogonadism. These agents appear hormonally neutral in healthy eugonadal men. The available evidence supports the concept that GLP-1 RAs act as metabolic–endocrine interventions that may partially restore HPG axis activity by improving the metabolic milieu, rather than functioning as direct testosterone-stimulating drugs. GLP-1 RAs should not be considered a substitute for testosterone replacement therapy in men with classical hypogonadism.

Preliminary pilot evidence further suggests that, in a distinct subgroup of men who remain persistently hypogonadal despite incretin-based therapy, adjunct testosterone may offer additional clinical benefit; however, this finding is based on a very small, non-randomised study and requires confirmation in larger controlled trials before informing routine clinical practice.

Future large-scale prospective trials are required to determine the durability of testosterone recovery, to clarify mechanisms underlying HPG-axis reactivation, and to define the optimal integration of incretin-based therapies—alone or in combination with TRT—with established endocrine treatments in men with metabolic hypogonadism [25].

## Figures and Tables

**Table 1:** Table of results for studies used in this review. Body weight change and insulin resistance (HOMA-IR) data: Graybill et al.—mean body weight change was not the primary focus; changes were not associated with weight loss magnitude. Lisco et al.—body weight change was not separately reported. La Vignera et al. (2025, tirzepatide)—significant body weight reduction over 2 months (see source paper for kg values). La Vignera et al. (2023, liraglutide)—body weight and composition changes were reported in the source paper. Lengsfeld et al.—minimal weight change was expected over the 4-week crossover. Grégorič et al.—significant body weight reduction with semaglutide over 24 weeks; no correlation was found between weight loss magnitude and testosterone change. Seminara et al.—late tirzepatide responders were defined by <5% body weight loss; TRT addition resulted in body fat reduction and lean body mass increase (+2.7 kg); HOMA-IR decreased significantly (2.9 ± 0.6 vs. 3.8 ± 0.7, *p* < 0.01 with TRT).

Study (Ref)	Study Design	Population	*n*	GLP-1 RA	Comparator	Duration	Hormones Measured	Direction of Change (↑/↔/↓)	Reproductive Outcomes	Key Results
Lengsfeld et al. (2024) [22]	Randomised double-blind placebo-controlled crossover RCT	Healthy eugonadal men	26 randomised; 24 completed	Dulaglutide	Placebo (crossover)	4 weeks per arm	Total testosterone LH FSH	Total T ↔ LH ↔ FSH ↔	Sexual desire Sexual function Semen parameters—all ↔	No significant effect on any reproductive hormone or semen parameter vs. placebo. Confirms GLP-1 RAs are endocrinologically neutral in healthy eugonadal men without metabolic disease.
Lisco et al. (2024) [23]	Retrospective cohort	Men with T2DM + erectile dysfunction	108 (45 metformin; 63 GLP-1 RA + metformin)	Long-acting GLP-1 RAs (mixed)	Metformin alone	12 months	Total testosterone Free testosterone	Total T ↑ Free T ↑ (vs ↓ in metformin-alone group)	Erectile function (IIEF)	Significant improvement in total and free testosterone vs. metformin alone. GLP-1 RA exposure was the strongest independent predictor of erectile function improvement.
Eid et al. (2025) [24]	Retrospective chart review	Men with obesity and/or type 2 diabetes prescribed GLP-1 RAs	53	GLP-1 receptor agonists (various; not specified individually)	Pre-treatment baseline (within-subject comparison)	Pre- vs post-therapy (duration not specified)	Total testosterone; Free testosterone	Total T ↑; Free T NR	Erectile function (SHIM); metabolic outcomes (weight, BMI, HbA1c)	Mean total testosterone increased by 111 ng/dL (*p* = 0.048). SHIM score improved by 2.4 points, but this was not statistically significant (*p* = 0.42). No association was found between weight change and testosterone change, suggesting testosterone improvements may be independent of weight loss
Graybill et al. (2021) [25]	Prospective cohort	Men with T2DM starting GLP-1 RA	51	Exenatide ER	Baseline (pre–post)	6 months	Total testosterone Free testosterone SHBG	Total T ↔ (overall) Total T ↑ in subgroup with baseline T < 320 ng/dL or significant HbA1c reduction	None reported	No significant overall testosterone change. Significant increases seen in hypogonadal subgroup or those with meaningful glycaemic improvement. Changes not associated with weight loss magnitude.
Gregorič et al. (2025) [26]	Randomised open-label trial	Obese men with T2DM and functional hypogonadism	25 (13 semaglutide; 12 TRT)	Semaglutide	Testosterone undecanoate (TRT)	24 weeks	Total testosterone	Total T ↑ (both groups)	Semen morphology	Semaglutide improved testosterone comparably to TRT. No correlation between weight loss and testosterone change. Semaglutide preserved semen morphology; TRT impaired it.
La Vignera et al. (2025) [27]	Controlled pilot study	Obese men with metabolic hypogonadism	83 (Group A 28; Group B 30; Group C 25)	Tirzepatide	Lifestyle/metformin control groups	2 months	Total testosterone Free testosterone Bioavailable testosterone LH FSH SHBG	Total T ↑ Free T ↑ Bioavailable T ↑ LH ↑ FSH ↑ SHBG ↑	Erectile function Body composition	Rapid increases in total, free, and bioavailable testosterone. Concurrent LH and FSH rises suggest HPG axis reactivation. Dual incretin agonism may amplify endocrine recovery.
La Vignera et al. (2023) [15]	Prospective interventional	Obese fertile men with functional hypogonadism	110 (Group B: liraglutide)	Liraglutide	Baseline; gonadotropins; TRT (other arms)	4 months	Total testosterone SHBG LH FSH	Total T ↑ SHBG ↑ LH ↑ FSH ↑	Semen parameters (concentration, motility, morphology) Erectile function (IIEF)	Significant testosterone and gonadotropin improvements. Semen parameter improvements comparable to gonadotropin therapy. Supports fertility-preserving role of GLP-1 RA in functional hypogonadism.
Seminara et al. (2026) [28]	Prospective pilot study	Obese hypogonadal late tirzepatide responders (<5% weight loss after ≥3 months)	10 (Group A: 5 tirzepatide alone; Group B: 5 tirzepatide + TRT)	Tirzepatide + testosterone undecanoate 1000 mg IM	Tirzepatide monotherapy	6 months	Total testosterone Free testosterone LH FSH SHBG Oestradiol (E2) HOMA-IR	Tirzepatide alone: Total T ↔, LBM ↓ Tirzepatide + TRT: Total T ↑, Free T ↑ LH ↑, FSH ↑ LBM ↑, HOMA-IR ↓	Erectile function (IIEF-5) Body composition (DXA) Physical activity (GPAQ)	Tirzepatide monotherapy failed to restore testosterone or sexual function. Addition of TRT improved LBM (+2.7 kg), IIEF-5, HOMA-IR, and nearly doubled physical activity. Preliminary only (*n* = 10, non-randomised).

**Table 2:** Randomised controlled trial(s)—RoB 2.

Study	Randomisation Process	Deviations from Intended Interventions	Missing Outcome Data	Measurement of Outcome	Selection of Reported Result	Overall Risk of Bias
Lengsfeld et al. (2024) [22]	Low	Low	Low	Low	Low	Low risk

**Table 3:** Non-randomised studies—ROBINS-I.

Study	Confounding	Selection of Participants	Classification of Intervention	Deviations from Intended Intervention	Missing Data	Outcome Measurement	Selective Reporting	Overall Risk of Bias
Lisco et al. (2024) [23]	Moderate	Low	Low	Low	Low	Low	Low	Moderate
Eid et al. (2025) [24]	Moderate	Moderate	Low	Low	Low	Moderate	Low	Moderate
Graybill et al. (2021) [25]	Moderate	Low	Low	Low	Low	Low	Low	Moderate
Gregorič et al. (2025) [26]	Moderate	Moderate	Low	Low	Low	Moderate	Low	Moderate
La Vignera et al. (2025) [27]	Serious	Moderate	Low	Moderate	Low	Moderate	Low	Serious
La Vignera et al. (2023) [15]	Serious	Moderate	Low	Moderate	Low	Moderate	Low	Serious
Seminara et al. (2026) [28]	Serious	Serious	Low	Moderate	Low	Moderate	Low	Serious

**Table 4:** GRADE summary.

Outcome	Studies Contributing	Study Design	Risk of Bias	Inconsistency	Indirectness	Imprecision	Publication Bias	Certainty of Evidence
Total testosterone change	7	RCT + observational	Serious	Moderate heterogeneity	Low	Serious (small samples)	Possible	Low
Free testosterone/SHBG	5	Observational	Serious	Moderate	Low	Serious	Possible	Low
Free testosterone/SHBG	4	Observational	Serious	Moderate	Moderate	Serious	Possible	Very low–Low
Semen parameters	3	Mixed	Serious	High heterogeneity	Moderate	Serious	Possible	Very low
Metabolic outcomes (HbA1c, weight)	7	Mixed	Moderate	Low–Moderate	Low	Moderate	Unlikely	Moderate

## Data Availability

No new data were created or analysed in this study. Data sharing is not applicable to this article.

## Supplementary Materials

The following supporting information can be downloaded at: https://www.mdpi.com/article/10.3390/cells15151319/s1.

## Abbreviations

The following abbreviations are used in this manuscript:

BMI	Body Mass Index
EAU	European Association of Urology
FSH	Follicle-Stimulating Hormone
GLP-1	Glucagon-Like Peptide-1
GLP-1 RA	Glucagon-Like Peptide-1 Receptor Agonist
GnRH	Gonadotropin-Releasing Hormone
HPG	Hypothalamic–Pituitary–Gonadal
HOMA-IR	Homeostatic Model Assessment of Insulin Resistance
ICS	International Continence Society
IIEF-5	International Index of Erectile Function (5-item)
LBM	Lean Body Mass
LH	Luteinising Hormone
MeSH	Medical Subject Headings
PRISMA	Preferred Reporting Items for Systematic Reviews and Meta-Analyses
RCT	Randomised Controlled Trial
RoB 2	Risk of Bias 2 Tool
ROBINS-I	Risk Of Bias In Non-randomised Studies of Interventions
SHBG	Sex Hormone-Binding Globulin
T2DM	Type 2 Diabetes Mellitus
TD	Testosterone Deficiency
TRT	Testosterone Replacement Therapy

## Appendix A. Full Search Strategy

The full electronic search strategy for PubMed/MEDLINE is presented below to ensure reproducibility. The PubMed search strategy was adapted appropriately for use in other databases, including the Cochrane Central Register of Controlled Trials (CENTRAL) and Google Scholar.

### Appendix A.1. PubMed/MEDLINE

The following search strategy was used:

(“Glucagon-Like Peptide 1 Receptor Agonists”[Mesh]
OR “GLP-1 receptor agonist*”
OR “GLP-1RA”
OR semaglutide
OR liraglutide
OR dulaglutide
OR exenatide
OR tirzepatide
OR “dual incretin”)

AND

(“Testosterone”[Mesh]
OR testosterone
OR “hypogonadism”[Mesh]
OR hypogonadism
OR “testosterone deficiency”
OR “functional hypogonadism”
OR “male reproductive hormones”
OR “gonadal function”)

AND

(“Spermatogenesis”[Mesh]
OR spermatogenesis
OR “semen parameters”
OR sperm
OR fertility
OR “reproductive function”
OR “erectile function”)

Filters applied:

Humans
Male
English language
Publication date: 1 January 2021 to 31 January 2026

### Appendix A.2. Cochrane Central Register of Controlled Trials (CENTRAL)

The PubMed search strategy was adapted for use in CENTRAL as follows:

(“GLP-1 receptor agonist*” OR semaglutide OR liraglutide
OR dulaglutide OR exenatide OR tirzepatide OR “dual incretin”)

AND

(testosterone OR hypogonadism OR “testosterone deficiency”
OR “male reproductive hormones”)

AND

(sperm OR spermatogenesis OR fertility
OR “semen parameters” OR “erectile function”)

### Appendix A.3. Google Scholar

Due to limitations in search functionality, a simplified search strategy was used:

(“GLP-1 receptor agonist” OR semaglutide OR liraglutide OR tirzepatide)
AND (testosterone OR hypogonadism OR “male reproductive hormones”)
AND (sperm OR fertility OR “erectile function”)

The first 200–300 results sorted by relevance were screened for eligibility. Additional studies were identified through citation tracking of relevant articles.

### Appendix A.4. Additional Search Methods

To maximise completeness of the evidence base:

Reference lists of all included studies and relevant review articles were manually screened.
Abstracts from major scientific meetings were reviewed, including:
Endocrine Society (ENDO)
European Association of Urology (EAU)
International Continence Society (ICS)
